# Alteration of Fecal Microbiota Profiles in Juvenile Idiopathic Arthritis. Associations with HLA-B27 Allele and Disease Status

**DOI:** 10.3389/fmicb.2016.01703

**Published:** 2016-10-26

**Authors:** Monica Di Paola, Duccio Cavalieri, Davide Albanese, Maddalena Sordo, Massimo Pindo, Claudio Donati, Ilaria Pagnini, Teresa Giani, Gabriele Simonini, Alessia Paladini, Paolo Lionetti, Carlotta De Filippo, Rolando Cimaz

**Affiliations:** ^1^Department of Neuroscience, Psychology, Drug Research and Child Health, Meyer Children’s Hospital, University of FlorenceFlorence, Italy; ^2^Department of Biology, University of FlorenceFlorence, Italy; ^3^Fondazione E. Mach, Research and Innovation CenterTrento, Italy; ^4^Rheumatology Unit, Anna Meyer Children’s Hospital, University of FlorenceFlorence, Italy; ^5^Institute of Biometeorology, National Research CouncilFlorence, Italy

**Keywords:** juvenile idiopathic arthritis, enthesitis-related arthritis, gut microbiota, HLA-B27 allele, metagenomics

## Abstract

Alteration of gut microbiota is involved in several chronic inflammatory and autoimmune diseases, including rheumatoid arthritis, and gut microbial “pro-arthritogenic” profiles have been hypothesized. Intestinal inflammation may be involved in spondyloarthropathies and in a subset of patients affected by Juvenile Idiopathic Arthritis (JIA), the most common chronic rheumatic disease of childhood. We compared the fecal microbiota composition of JIA patients with healthy subjects (HS), evaluating differences in microbial profiles between sub-categories of JIA, such as enthesitis-related arthritis (JIA-ERA), in which inflammation of entheses occurs, and polyarticular JIA, non-enthesitis related arthritis (JIA-nERA). Through taxon-level analysis, we discovered alteration of fecal microbiota components that could be involved in subclinical gut inflammation, and promotion of joint inflammation. We observed abundance in *Ruminococcaceae* in both JIA categories, reduction in *Clostridiaceae* and *Peptostreptococcaceae* in JIA-ERA, and increase in *Veillonellaceae* in JIA-nERA, respectively, compared with HS. Among the more relevant genera, we found an increase in *Clostridium cluster XIVb*, involved in colitis and arthritis, in JIA-ERA patients compared with HS, and a trend of decrease in *Faecalibacterium*, known for anti-inflammatory properties, in JIA-nERA compared with JIA-ERA and HS. Differential abundant taxa identified JIA patients for the HLA-B27 allele, including *Bilophila, Clostridium cluster XIVb, Oscillibacter*, and *Parvimonas*. Prediction analysis of metabolic functions showed that JIA-ERA metagenome was differentially enriched in bacterial functions related to cell motility and chemotaxis, suggesting selection of potential virulence traits. We also discovered differential microbial profiles and intra-group variability among active disease and remission, suggesting instability of microbial ecosystem in autoimmune diseases with respect to healthy status. Similarly to other chronic autoimmune and inflammatory diseases, different microbial profiles, as observed among different JIA subgroups compared to HS, and potential functional acquisition related to migration, could promote inflammation and contribute to the disease pathogenesis.

## Introduction

Characterization of bacterial commensal communities in autoimmune and inflammatory diseases is a topic of great interest for understanding the role and interaction of intestinal microbiota with the host immune system.

It is known that the gut microbiota is shaped by several environmental factors, including dietary habits, antibiotics, infectious agents, and air pollution (De Filippo et al., 2010; David et al., 2014a; Salim et al., 2014), and in turn, that microbiota shapes the immune system, modulating homeostasis in healthy status individuals or promoting inflammation when dysbiosis occurs. Recent findings demonstrate that alteration in the equilibrium among commensal bacteria is associated not only with Inflammatory bowel disease (IBD), allergy, diabetes and celiac disease (Cheng et al., 2013), but also with rheumatoid arthritis (Vaahtovuo et al., 2008; Sandhya et al., 2016; Scher et al., 2016).

Juvenile idiopathic arthritis (JIA) is the most common chronic rheumatic disease in children, and comprises a clinically heterogeneous group of conditions characterized by chronic arthritis, synovial inflammation and erosion of bone and cartilage (Ravelli and Martini, 2007). According to several factors, including the number of affected joints, JIA is divided in psoriatic, oligoarticular (up to four affected joints), polyarticular (five or more affected joints) (Oberle et al., 2014), and enthesitis-related arthritis (ERA), in which entheses (attachments of tendons and ligaments to bone) are affected. ERA accounts for 10–20% of JIA and is considered the equivalent of spondyloarthropathy, a disease frequently characterized by clinical and subclinical intestinal involvement (Bryan and Rabinovich, 2014; Oberle et al., 2014; Aggarwal and Misra, 2015).

Immunological, genetic, and environmental factors are involved in the pathogenesis of ERA (Stoll et al., 2014; Gmuca and Weiss, 2015). Gene variants in the Major Histocompatibility Complex, especially the HLA-B27 alleles, have been identified as predisposing factors (Gmuca and Weiss, 2015).

Alterations of gut microbiota (dysbiosis) and a decrease in gut microbiota richness (Li et al., 2014; Collado et al., 2015; Rogers, 2015) are emerging as factors associated with the development of inflammatory and systemic autoimmune diseases (Yeoh et al., 2013; Longman and Littman, 2015). Of great interest is the understanding of dysbiosis as a trigger or a reflection of autoimmune and inflammatory disorders (Chung and Kasper, 2010; Stoll et al., 2014), in fact, also autoimmunity can drive instability of the gut microbial ecosystem.

Studies in germ-free animal models reveal relationships between microorganisms, mucosal immunity, and joint inflammation (Taurog et al., 1994; Rath et al., 1996; Longman and Littman, 2015). Recent studies in humans suggest that alteration of oral and gut microbiota and an increase in leaky gut could trigger systemic joint inflammation in the context of pre-existent autoimmunity (Scher et al., 2013, 2015; Brusca et al., 2014; Costello et al., 2014; Taneja, 2014; Longman and Littman, 2015; Zhang et al., 2015).

However, although arthritis susceptibility has been linked to the gut microbiome, and it has proposed that in synovial fluids the presence of pro-arthritogenic bacterial DNA, deriving by circulating intestinal bacterial products, may promote synovial inflammation (Kempsell et al., 2000; Gerard et al., 2001; Moen et al., 2006; Oberle et al., 2014), a causal relationship between bacterial infection and onset of rheumatological diseases has not yet been firmly demonstrated. Further studies on leaky gut syndrome could clarify the presence of bacterial products circulating and influencing the systemic immune response, also in light of the presence of bacteria in non-rheumatoid arthritis controls (Kempsell et al., 2000), and of potential microbial contamination found in amplification technique of 16S rDNA, as previous observed (Grahn et al., 2003).

A targeted-metagenomics approach can provide an in-depth characterization of microbial communities, allowing investigation of correlations between microbiota composition and human pathologies (Lozupone et al., 2012).

In this study, we characterized and compared the gut microbiota of JIA patients, affected by enthesitis-related arthritis (JIA-ERA) and polyarticular JIA (non-enthesitis-related arthritis, nERA), with healthy subjects (HS), in order to define specific microbial “pro-arthritogenic” profiles.

## Materials and Methods

### Sampling of Subjects

We enrolled 29 JIA patients (13 males and 16 females, age range 2–18 years), 19 of whom were affected by ERA and 10 by polyarticular JIA (nERA). Exclusion criteria were: acute diarrhea, infectious gastroenteritis, antibiotic treatment in the previous 3 months, and diagnosis of chronic gastrointestinal disease. A total of 29 healthy children and adolescents (11 males and 18 females; age range 2–18 years) not affected by autoimmune and inflammatory conditions, infectious gastroenteritis, or chronic gastrointestinal disease, were enrolled as controls.

We collected a fecal sample from each subject, and a second one from 17 ERA patients three months later, in order to evaluate microbiota variability. We also gathered clinical information, including: enthesitis and arthritis localization, age of onset, HLA-B27 status, family history of ankylosing spondylitis or other HLA-B27-related diseases, comorbidities, laboratory parameters (C Reactive Protein, Erythrocyte Sedimentation Rate, ANA and pANCA autoantibody positivity), and pharmacological treatments (**Table 1**; Supplementary Tables S1 and S2). None of the patients underwent treatment with proton pump inhibitors (PPIs). Active disease was defined by the presence of active arthritis and/or enthesitis at the time of stool sampling. As reported in **Table 1**, a total of four JIA-ERA and four JIA-nERA patients had clinically active disease. Fecal calprotectin (Aomatsu et al., 2011) was also assessed by ELISA assay for subclinical intestinal inflammation (Eurospital, Trieste, Italy). Parents or guardians gave written informed consent for fecal samples and clinical data collection of their children. The study protocol was approved by the Ethics Committee of the Meyer Children’s Hospital, Florence, Italy (Protocol ref. Nov 12th, 2013), and carried out in accordance with the approved guidelines.

**Table 1:** Clinical features of JIA patients and age/gender information of healthy subjects (HS).

	ERA	nERA	HS
Number	19	10	29
Age (years) median, range	14.3; 9 to 18	10.5; 2 to 17	13; 2 to 18
Male: Female, number	13:6	0:10	11:18
Disease duration (months) median, range	55; 2 to 93	97; 3 to 150	–
Acute disease, number (%)	4 (21%)	4 (40%)	–
Calprotectin positive, number (%)	2 (10.5%)	2 (20%)	–
High Erythrocyte sedimentation rate, number (%)	3 (15.7%)	0 (0%)	–
HLA-B27+	9 (47%)	0 (0%)	–
ANA+	4 (21%)	9 (90%)	–

Complications or comorbidities, number (%)

No	10 (52.6%)	3 (30%)	–
Uveitis	1 (5.2%)	7 (70%)	–
β- thalassemia	1 (5.2%)	0 (0%)	–
Pectum escavatum	2 (10.5%)	0 (0%)	–
Asthma	2 (10.5%)	0 (0%)	–
Osteoporosis	1 (5.2%)	0 (0%)	–
Obesity	1 (5.2%)	0 (0%)	–
Hypothyroidism	1 (5.2%)	0 (0%)	–
Epigastric hernia	1 (5.2%)	0 (0%)	–
Cataract	1 (5.2%)	0 (0%)	–
Breast fibroadenoma	1 (5.2%)	0 (0%)	–

Treatment, number of patients (%)

NSAIDs	18 (95%)	1 (10%)	–
Sulfasalazine	8 (42%)	0 (0%)	–
Steroids	4 (21%)	0 (0%)	–
Methotrexate	4 (21%)	0 (0%)	–
Etanercept	3 (15.7%)	4 (40%)	–
Abatacept	1 (5.2%)	5 (50%)	–
Adalimumab	2 (10.5%)	0 (0%)	–

*HLA, Human leukocyte antigen; ANA, Anti-nuclear antibody; NSAIDs, Non-steroidal anti-inflammatory drugs.*

### DNA Extraction

Fecal samples were preserved in RNAlater (Qiagen) at 4°C for the first 48 h, and kept at -80°C until DNA extraction. Bacterial genomic DNA extraction and quality control were carried out following our previous protocol (De Filippo et al., 2010).

### Pyrosequencing

For each sample, we amplified the 16S rRNA gene using the special fusion primer set specific for V5-V6 hypervariable regions and corresponding to primers 784F and 1061R described by Andersson et al. (2008), and using the FastStart High Fidelity PCR system (Roche Life Science, Milano, Italy). The 454 pyrosequencing was carried out on the GS FLX+ system using the XL+chemistry following the manufacturer recommendations (details in Supplementary Materials).

### Data Analysis

Pyrosequencing resulted in a total of 2,180,826 16S rDNA reads with a mean of 29,078 sequences per sample. Average sequence lengths were 290 nt (±SD 45) and 286 nt (±SD 50) for the first and second run, respectively. Raw 454 files were demultiplexed using Roche’s.sff file software, and available at the European Nucleotide Archive^1^ under the accession study ERP013262. Reads were pre-processed using the MICCA pipeline (version 0.1)^2^ (Albanese et al., 2015). Forward and reverse primer trimming and quality filtering were performed using micca-preproc truncating reads shorter than 280nt (quality threshold = 18). *De novo* sequence clustering, chimera filtering and taxonomy assignment were performed by micca-otu-denovo (parameters -s 0.97 -c). Operational Taxonomic Units (OTUs) were assigned by clustering the sequences with a threshold of 97% pair-wise identity, and their representative sequences were classified using the RDP software version 2.7 (Wang et al., 2007). Template-guided multiple sequence alignment was performed using PyNAST57 (version 0.1) (Caporaso et al., 2010) against the multiple alignment of the Greengenes 16S rRNA gene database (DeSantis et al., 2006) filtered at 97% similarity. Finally, a phylogenetic tree was inferred using FastTree (Price et al., 2010) and micca-phylogeny (parameters: -a template-template-min-perc 50). Sampling heterogeneity was reduced by rarefaction, obtaining 12,964 sequences per sample.

Chao1 index and Shannon entropy (indicators of alpha diversity) and UniFrac (Lozupone and Knight, 2005) and Bray–Curtis dissimilarities (indicators of beta diversity) were calculated using the phyloseq package (McMurdie and Holmes, 2014) of the R software suite. Exploratory analysis was performed by Principal coordinates analysis (PCoA) using the phyloseq package of the R software suite. Multiple-rarefaction PCoA plots (“jackknifed” PCoA plots) (Lozupone et al., 2011) were computed to assess the robustness of the beta-diversity analyses.

The significance of between-groups differentiation on the UniFrac distances and Bray–Curtis dissimilarity was assessed by PERMANOVA using the adonis() function of the R package vegan with 999 permutations.

As a measure of species evenness, we calculated Dominance (1-Simpson index) and Equitability (Shannon diversity index divided by the logarithm of taxa number) by using PAST v 3.12 (Hammer et al., 2001).

To compare the relative abundances of OTUs among the three groups of subjects, the two-sided, unpaired Wilcoxon test was computed, removing taxa not having a relative abundance of at least 0.1%, in at least 20% of the samples, and using the function mt() in the phyloseq library and the *p*-values were adjusted for multiple comparison controlling the family wise Type I error rate (minP procedure). Further significant differences in bacterial taxa were supported by non-parametric White’s test and Welch’s test by using STAMP (Parks et al., 2014), and *p*-values were adjusted for multiple comparisons by Storey FDR, as proposed in STAMP.

On the basis of the relative abundances, the metagenomic biomarker discovery and related statistical significance were assessed using the linear discriminant analysis (LDA) effect size (LEfSe) method (Segata et al., 2011). LEfSe uses the Kruskal–Wallis rank-sum test to identify features with significantly different abundances between assigned taxa compared to the groups, and LDA to estimate the size effect of each feature. An alpha significance level of 0.05, either for the factorial Kruskal–Wallis test among classes or for the pairwise Wilcoxon test between subclasses, was used. A size-effect threshold of 2.0 on the logarithmic LDA score was used for discriminative microbial biomarkers.

In order to predict how taxonomic differences between fecal microbiota of the three groups impact their microbial metabolic potential, we applied PICRUSt (Phylogenetic Investigation of Communities by Reconstruction of Unobserved States) (Langille et al., 2013), a computational approach useful to infer the functional contribution of microbial communities on 16S rDNA sequencing data set. PICRUSt implements an extended ancestral-state reconstruction algorithm to predict which gene families are present, and then combines gene families to estimate the significant differences in the main functional classes (KEGG categories) of the composite metagenome. From a OTUs table with associated Greengenes identifiers, we obtained the final output from metagenome prediction as an annotated table of predicted gene family counts for each sample, where the encoded functions of each gene family are orthologous groups or other identifiers such as KEGG orthologs (KOs). The functional pathways discovery and related statistical significance were assessed by LEfSe.

## Results

### Clinical Features of JIA Patients

We collected clinical features, as well as comorbidities and therapies, for each enrolled patient at the time of fecal sampling (**Table 1**; more details in Supplementary Tables S1 and S2). As known, due to the two different subsets of JIA, females are affected from polyarticular JIA two to four times more often than males (Oberle et al., 2014). In our cohort, most of the enrolled JIA-ERA patients were males (13/19), while all JIA-nERA patients were females (10/10). Thus, we evaluated the sex/gender as a potential variable influencing the fecal microbiota composition in both JIA categories. Regarding HLA-B27 status, 47% (9/19) of JIA-ERA patients resulted HLA-B27 positive, while all JIA-nERA patients were HLA-B27 negative. Calprotectin level, a measurement of intestinal inflammation, was positive (>100 μg/g) in 10.5% (2/19) of JIA-ERA and in 20% (2/10) of JIA-nERA patients (see Materials and Methods).

### Microbiota Characterization by 16S rDNA Sequencing in the JIA Groups and Healthy Controls

We sequenced the V5–V6 hypervariable region of 16S rRNA gene for the meta-taxonomic study of microbiota in a total of 75 fecal samples from 19 JIA-ERA patients, 10 JIA-nERA, and 29 HS. From 17 JIA-ERA patients a second fecal sample was collected 3 months later, to evaluate microbiota variability over time (see Materials and Methods).

**FIGURE 1:**
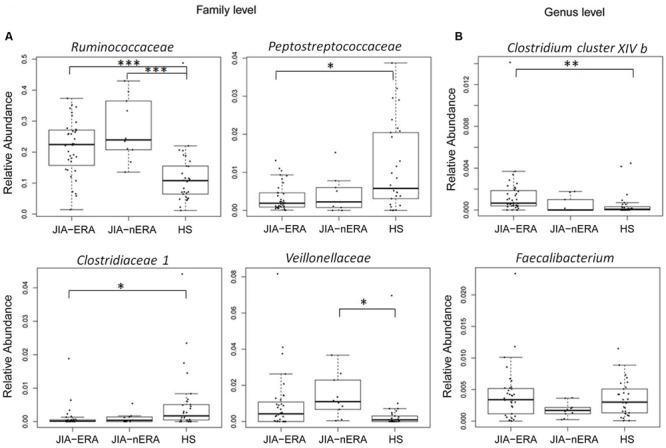
**Relative abundances of fecal bacterial components in JIA and HS groups.** Box plot of statistically significant different bacterial **(A)** families and **(B)** genera in JIA patients compared to HS (Pairwise comparisons using Wilcoxon rank sum test; ^∗∗∗^*p* < 0.001; ^∗∗^*p* < 0.01; ^∗^*p* < 0.05 FDR adjusted). For *Faecalibacterium* genus, statistical significance of the comparison JIA-ERA vs. JIA-nERA was obtained by Welch’s test without multiple testing correction.

The taxonomic distribution in the three groups showed variations in gut microbiota composition (**Figure 1**; Supplementary Figure S1). Firmicutes, the dominant phylum of gut microbiota, was the most abundant in all samples (over 50% of total reads; Supplementary Figure S1A). Considering the 20 most abundant families (Supplementary Figure S1B), we found statistically significant differences between groups in *Ruminococcaceae, Peptostreptococcaceae, Clostridiaceae I* and *Veillonellaceae* (**Figure 1A**). *Ruminococcaceae* was more abundant in children in both JIA categories, compared with HS (21.6% in JIA-ERA and 27.2% in JIA-nERA vs. 12.0% in HS; Wilcoxon rank-sum test, JIA-ERA vs. HS *p* = 0.0004; JIA-nERA vs. HS *p* = 0.0006; **Figure 1A**). Although there was a reduction of *Peptostreptococcaceae* and *Clostridiaceae I* in all JIA patients (ERA and nERA) compared with HS (0.3% in JIA-ERA, 0.4% in JIA-nERA and 1.1% in HS; 0.1% in JIA vs. 0.5% in HS, respectively), we found statistically significant differences only between JIA-ERA vs. HS (Wilcoxon rank-sum test, *p* = 0.033 for *Peptostreptococcaceae, p* = 0.017 for *Clostridiaceae I*, respectively, **Figure 1A**). We observed a statistically significant predominance of *Veillonellaceae* in JIA-nERA compared with HS (1.4% in JIA-nERA vs. 0.4% in HS; Wilcoxon rank-sum test, *p* = 0.012; **Figure 1A**).

Considering gender as potential variable influencing the gut microbiota composition, we confirmed that among the enrolled female subjects (6 JIA-ERA, 10 JIA-nERA, and 18 HS), *Firmicutes* were more abundant in the JIA-nERA group compared with HS (Supplementary Figure S2A; Wilcoxon rank-sum test *p* < 0.05). Among the minor constituents of fecal microbiota, we observed an increase in *Sutterellaceae* and *Enterobacteriaceae* in JIA-ERA female patients compared with female HS (2.5% in JIA-ERA vs. 1.2% in HS and 0.5% in JIA-ERA vs. 0.3% in HS, respectively; Wilcoxon rank-sum test, *p* < 0.05; Supplementary Figure S2B), and *Streptococcaceae* in JIA-nERA compared with HS (1% in JIA-nERA vs. 0.3% in HS; Wilcoxon rank-sum test, *p* < 0.05; Supplementary Figure S2B).

At genus level, we found an abundance of *Clostridium cluster XIVb* in JIA-ERA patients compared with HS (0.23% in JIA-ERA vs. 0.1% in HS; Wilcoxon rank-sum test, *p* = 0.007; **Figure 1B**). Moreover, we observed a decrease in the relative abundance in *Faecalibacterium* in JIA-nERA compared with either JIA-ERA or HS, even if not statistically significant (0.18% in JIA-nERA vs. 0.35% in HS; 0.18% in JIA-nERA vs. 0.41% in JIA-ERA; **Figure 1B**).

**FIGURE 2:**
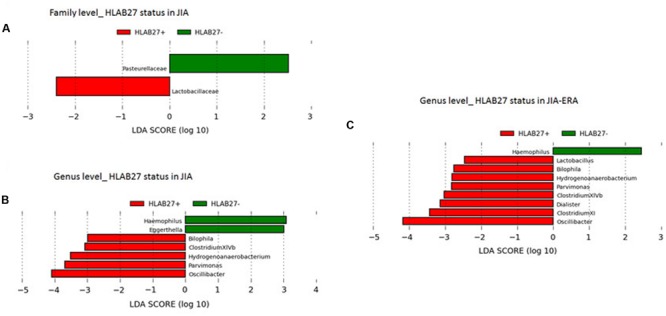
**Differences in bacterial taxa related to HLA-B27 status.** LEfSe analysis shows a statistically significant enrichment of **(A)** families, and **(B,C)** genera, in **(A,B)** all JIA patients and in **(C)** JIA-ERA patients, considering the HLA-B27 status. LEfSe results indicate a sequentially significant ranking among groups (Alpha value = 0.05 for the factorial Kruskal–Wallis test among classes). The threshold for the logarithmic LDA score was 2.0.

By LDA Effect Size (LEfSe; see Materials and Methods), we evaluated significant differences in abundance between assigned taxa with respect to JIA patient groups. We observed differentially abundant taxa discriminating for HLA-B27 status. At family level, increased *Lactobacillaceae* in HLA-B27 positive-JIA patients, and *Pasturellaceae* in HLA-B27 negative-JIA patients were found (**Figure 2A**). Of the increased genera in HLA-B27 positive JIA patients, we observed *Bilophila, Parvimonas*, and *Oscillibacter*, while *Haemophilus* and *Eggerthella*, were differentially enriched in HLA-B27 negative patients (**Figure 2B**). When considering only the JIA-ERA group, in addition to the five genera reported in **Figure 2B**, *Lactobacillus, Clostridium cluster XI*, and *Dialister* were enriched in HLA-B27 positive patients, while only *Haemophilus* discriminated for HLA-B27 negative status (**Figure 2C**).

Further, we evaluated correlations between microbiota profiles of JIA patients and fecal calprotectin, as well as with respect to different medical treatments. We did not find any significant correlation between fecal calprotectin levels and microbiota profiles (Supplementary Tables S1 and S2), probably due to the low number of patients with concomitant intestinal inflammation at the time of sampling. Regarding the effect of pharmacological therapies on gut microbiota of JIA patients, in our cohorts, JIA-ERA patients were treated with NSAIDs, alone or associated with sulfasalazine/methotrexate/biologics in different combinations, as reported in **Table 1** and Supplementary Table S1. JIA-nERA patients were mainly treated with biologic drugs, such as Abatacept and Etanercept (**Table 1**; Supplementary Table S2). Despite the low number of patients stratified by pharmacological treatment, LEfSe analysis showed indications of association among different bacterial profiles and therapies (as reported in Supplementary data and Supplementary Figures S3A–C), among which we observed enrichment in *Collinsella*, associated with exacerbation of joint disease (Chen et al., 2016), in JIA-ERA patients treated with combined NSAIDs and sulfasalazine therapy.

However, a larger cohort of patients would be needed to strengthen our preliminary results and to understand the causality between therapies, gut microbiota profiles and clinical status.

**FIGURE 3:**
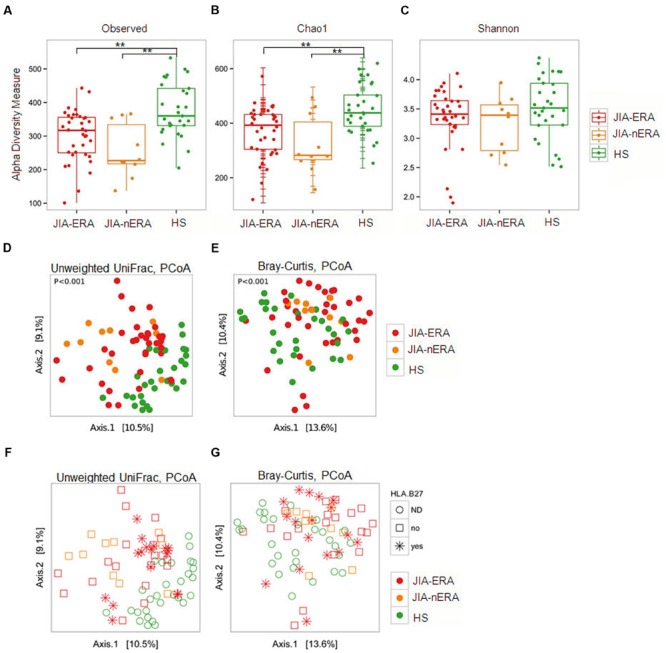
**Alpha and beta diversity measures. (A–C)** Box plots of **(A)** number of observed OTUs, **(B)** Chao 1, and **(C)** Shannon indexes in the three studied populations. Pairwise comparisons using the Wilcoxon rank sum test., ^∗∗^*p*-value < 0.005, false discovery rate adjustment. **(D,E)** PCoA of **(D)** unweighted UniFrac and **(E)** Bray–Curtis dissimilarities. **(F,G)** PCoA of unweighted UniFrac and Bray–Curtis dissimilarities considering HLAB27 status (red asterisks). Colored dots: red = JIA-ERA, orange = JIA-nERA, and green = HS samples.

In order to evaluate differences in the microbial biodiversity (alpha-diversity) among groups, we calculated the observed number of OTUs (a measurement of the total number of species present in a microbial community) and the Chao1 index (an indicator of species richness based on number of rare species), observing a significant reduction in alpha diversity in the JIA samples compared to HS (**Figures 3A,B**; *p*-value < 0.005 JIA-ERA vs. HS and JIA-nERA vs. HS; Supplementary Table S3). This suggests that microbiota of JIA patients is associated with biodiversity depletion, as observed in IBD patients. However, we did not find significant differences among the three groups using measures of biodiversity that take the evenness of the species distributions into account, like the Shannon entropy (**Figure 3C**), the Dominance (1-Simpson index) and Equitability (Pielou index; Materials and Methods; Supplementary Figure S4).

To estimate the variability of microbial communities between-sample (beta-diversity), we calculated the Bray-Curtis and unweighted UniFrac dissimilarities (see Materials and Methods). PCoA on unweighted UniFrac dissimilarities showed that both JIA samples (ERA and nERA) were more similar to each other than to HS samples (**Figure 3D**). Bray Curtis dissimilarity confirmed the differences among samples in the three populations (PERMANOVA, *p* < 0.001; **Figure 3E**). Considering HLA-B27 status, PCoA, calculated on unweighted UniFrac and Bray–Curtis dissimilarity, showed that HLA-B27 positive JIA-ERA patients form subgroups with respect to other JIA-ERA samples (**Figures 3F,G**), confirmed also by neighbor joining clustering based on Bray–Curtis dissimilarities (Supplementary Figure S5). However, the sample distribution of patients and HS was more marked based on disease and health status (**Figures 3D,E**).

### Fecal Microbiota Comparison between Acute Disease and Remission in JIA Categories

**FIGURE 4:**
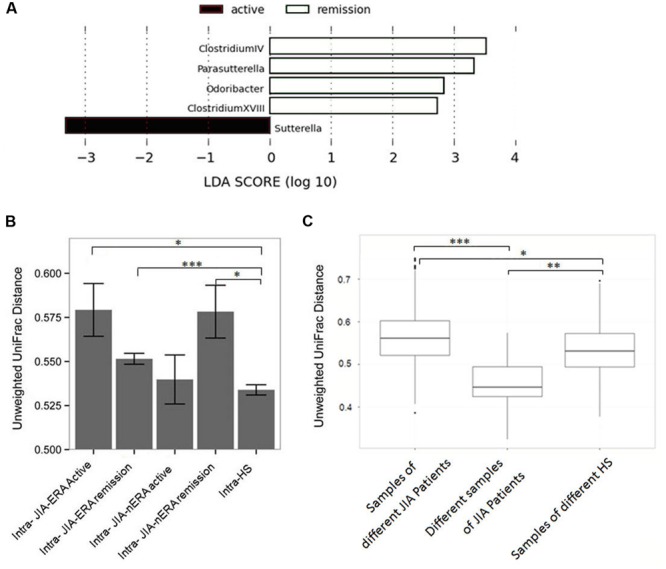
**Fecal microbiota comparison in patients during acute disease and in remission. (A)** LEfSe analysis shows a statistically significant enrichment of bacterial genera in JIA patients, in active disease (black) and in remission (white). LEfSe results indicate significant ranking among groups (Alpha value = 0.05 for the factorial Kruskal-Wallis test among classes). The threshold for the logarithmic LDA score was 2.0. **(B)** Intra-group distances calculated by Unweighted UniFrac between samples collected during active disease and in remission from JIA-ERA and JIA-nERA patients. *P*-values displayed are the results of Wilcoxon rank-sum tests. **(C)** Box plots of relative distances, calculated by Unweighted UniFrac, among different JIA samples and HS samples and distances of different the two samples of the same JIA-ERA patient. *P*-values displayed are the results of Wilcoxon rank sum test; ^∗^*p*-value < 0.05, ^∗∗^*p*-value < 0.01; ^∗∗∗^*p*-value < 0.001.

At the moment of fecal sampling, 21% of JIA-ERA patients (4/19) and 40% of JIA-nERA patients (4/10) had clinically active disease. Despite the reduced number of samples, LEfSe analysis showed significant differences in abundances of bacterial genera in samples collected during clinically active disease and during remission. In particular, *Sutterella* was increased in samples collected during active disease, while *Clostridium cluster IV* and *XVIII, Parasutterella* and *Odoribacter* were enriched in samples collected from patients in remission (**Figure 4A**).

Furthermore, we calculated intra-group distances by unweighted UniFrac, comparing all JIA samples collected from patients with active disease and those in remission, versus the HS samples. Although differences were not statistically significantly, we observed a trend indicating higher intra-group distances between JIA-ERA samples collected during active disease with respect to intra-group distances between JIA-nERA during active disease (**Figure 4B**; intra-active JIA-ERA vs. intra-active JIA-nERA; Supplementary Table S4). Within the JIA-ERA group, we found more variation in microbiota profile in samples collected during active disease compared to remission (**Figure 4B**; intra-active JIA-ERA vs. intra-remission JIA-ERA; Supplementary Table S4). On the contrary, although not statistically significantly different, the intra-distance of active disease samples in the JIA-nERA group was lower than in remission samples (**Figure 4B**; intra-active JIA-nERA vs. intra-remission JIA-nERA; Supplementary Table S4), indicating different microbial heterogeneity in active disease and remission in the two different JIA subsets. Comparing the samples collected from patients in remission, we observed that distances within the JIA-nERA group were greater than within the JIA-ERA group (**Figure 4B**; intra-remission JIA-nERA vs. intra-remission JIA-ERA; Supplementary Table S4).

Interestingly, when we compared JIA groups with HS, the intra-group distance in the HS group was lower than within both JIA groups (**Figure 4B**) and significantly correlated with intra-distances observed in JIA-ERA patients, either active or in remission, and with intra-distance observed in JIA-nERA patients in remission (Wilcoxon rank-sum test; intra-HS vs. intra-active JIA-ERA *p* = 0.02; intra-HS vs. intra-remission JIA-ERA *p* = 0.0004; intra-HS vs. intra-remission JIA-nERA *p* = 0.03; Supplementary Table S4), indicating that the microbiota composition within the healthy group was significantly more homogeneous than those within the JIA groups.

Next, we evaluated microbiota variations over time in patients of the JIA-ERA group, considering pairwise UniFrac distances between samples A and B (collected three months apart; see Materials and Methods). The matrices obtained by PCoA, derived from pairwise unweighted and weighted UniFrac distances between samples A vs. B, allowed us to explore inter- and intra-individual similarities or dissimilarities among samples, showing in most part of the cases lower distances between samples of the same patient than from different patients (Supplementary Figure S6). Comparing the distributions among all JIA and HS samples vs. the JIA-ERA samples at two different times by Unweighted UniFrac mean distances, we confirmed that samples from the same patient are more similar than samples from different patients, as well as samples from HS, as expected (**Figure 4C**).

### Metabolic Function Prediction

**FIGURE 5:**
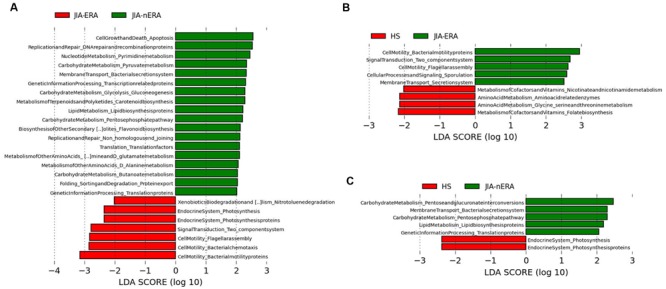
**Metabolic function prediction associated to the microbiota profiles.** LEfSe analysis, performed on metabolic functions inferred by PICRUSt analysis shows statistically significant enrichment of KEGG categories in **(A)** JIA-ERA vs. JIA-nERA, **(B)** JIA-ERA vs. HS, and **(C)** in JIA-nERA vs. HS. LEfSe results indicate significant ranking among groups (Alpha value = 0.05 for the factorial Kruskal–Wallis test among classes). The threshold for the logarithmic LDA score was 2.0.

The microbiota is able to affect host physiology and metabolic functions, contributing to normal development, and homeostasis of the immune system in the gut, modulating epithelial cell proliferation, and protecting against pathogenic bacteria (Tremaroli and Backhed, 2012; Sommer and Backhed, 2013). Bacterial species are known to carry and transfer operons containing genes for different metabolic functions. Different bacterial species are enriched for certain functions and these correlations have been categorized in well-organized databases (Kanehisa et al., 2014). Therefore, in order to clarify how phylogenetic differences between the fecal microbiota of JIA patients impact their metabolic potential, we applied PICRUSt (Phylogenetic Investigation of Communities by Reconstruction of Unobserved States), a computational approach used to predict the functional composition of a metagenome (Langille et al., 2013). LEfSe analysis performed on PICRUSt output (Supplementary Table S5) showed differentially enriched functional classes (KEGG categories) among JIA subgroups compared with HS (**Figure 5**). Within the main KEGG categories, we observed significant enrichment of functions related to cell motility (Flagellar assembly, Ko:02040, Bacterial chemotaxis and Bacterial motility proteins, Ko:02030) in JIA-ERA compared with JIA-nERA and HS (**Figures 5A,B**). Pathways related to Membrane transport (Secretion system, Ko:03070) and unclassified Cellular Processes and Signaling (Sporulation) were significantly enriched, respectively, in JIA-ERA and JIA-nERA compared with HS group (**Figures 5B,C**). A remarkable enrichment in metabolic functions regarding carbohydrate metabolism, lipid metabolism, aminoacid metabolism, and other aminoacid metabolism identified a core of metabolic capabilities, especially in JIA-nERA metagenome compared to JIA-ERA (**Figure 5A**) and HS (**Figure 5C**). On the other hand, metabolism of cofactors and vitamins (folate biosynthesis and nicotinate and nicotinamide metabolism) and metabolism of other aminoacids (Glycine, Serine, Threonine) significantly characterized HS metagenome compared to JIA-ERA (**Figure 5B**).

## Discussion

There are many lines of evidence that link the microbiota to rheumatic diseases. Animal models have been used to establish this possible correlation, such as the use of germ-free and gnotobiotic mice, in which animals were colonized with a specific microbial population, or through the use of antibiotics to understand the effect of microbiota modulation on rheumatic diseases. Principal limits of animal studies are the sample size and the difficulty to mimic the complex multifactorial pathogenesis of these pathologies.

In our study, we tried to define the role of microbiota in patients affected by JIA. Alterations and decrease of microbiota richness were recently found in JIA compared with healthy controls (Stoll et al., 2014), as well as in rheumatoid arthritis, ankylosing spondylitis and psoriatic arthritis, the latter two conditions related to spondyloarthropathies (Stebbings et al., 2002; Scher et al., 2013, 2015; Costello et al., 2014; Gill et al., 2015). We also observed variations in fecal microbiota composition and a reduction of microbial richness among JIA patients, affected by ERA and polyarticular JIA (nERA), in comparison with HS.

When compared to HS, in both JIA categories, we found statistically significant abundance in *Ruminococcaceae*, reduction in *Clostridiaceae* and *Peptostreptococcaceae* in JIA-ERA, and increase in *Veillonellaceae* in JIA-nERA. Of note, abundance in *Veillonellaceae* was recently found associated to ankylosing spondylitis (Costello et al., 2014). Conversely to our results, previous studies on ankylosing spondylitis (Costello et al., 2014; Scher et al., 2015) and on oligoarticular and polyarticular JIA (Tejesvi et al., 2016) showed depletion of either *Ruminococcaceae* or *Veillonellaceae*.

The enrichment of anaerobic Gram-positive *Clostridium cluster XIVb* in JIA-ERA patients suggests a causal relation with inflammation. In fact, members of *cluster XIVb, C. propionicum*, and *C. colinum* (Collins et al., 1994) were previously observed in poultry ulcerative enteritis (Berkhoff, 1985). Also, studies in animal models have indicated that cell wall peptidoglycans of *Clostridium* and other anaerobic Gram-positive species can induce either chronic and erosive or transient arthritis (Severijnen et al., 1989; Simelyte et al., 2003). The observed decreasing trend in *Faecalibacterium* in JIA-nERA, considered to be an anti-inflammatory microorganism and a marker of health, has also been consistently reported in Crohn’s disease patients (Sokol et al., 2008). Conversely to our results, a recent study showed reduction of *Faecalibacterium prasnutzii* in JIA-ERA patients (Stoll et al., 2014).

The partial agreement of our findings with results obtained in other studies could be due to several factors, including variabilities and size of cohorts (different JIA categories, disease status, untreated, or treated patients), as well as geography, environment or dietary habits of the patients, as shown by David and co-workers (David et al., 2014b).

Despite the limited possibility to generalize our results, due to the reduced number of patients in the cohorts, our results on JIA show intriguing links in terms of fecal microbiota profiles, with IBD and other autoimmune diseases associated to gastrointestinal disorders.

We are well aware that the known predominance of females in JIA-nERA introduces a potential gender effect, yet *Enterobacteriaceae* and *Streptococcaceae*, enriched in JIA-ERA and in JIA-nERA female patients, were also found to be correlated with intestinal inflammation as observed in biopsy samples of Crohn’s disease patients (Gevers et al., 2014) and the increase in *Sutterellaceae* observed in JIA-ERA female patients, as well as in samples collected in active disease, is also in line with increase in *Sutterella* previously found in children with autism suffering from gastrointestinal disorder (Williams et al., 2012). Moreover, in samples collected in remission, we found abundance in *Clostridium* spp. *cluster IV* that have been reported to be inducers of colonic T regulatory cell (Atarashi et al., 2011), as well as in *Odoribacter*, known producer of Short Chain Fatty Acids (SCFAs), anti-inflammatory metabolites, and previously found reduced in IBD (Morgan et al., 2012). However, in remission we also found enriched bacteria involved in intestinal inflammation and metabolic disorders, such as *Parasutterella*, found in a mice model of chemically induced colitis (Zhang et al., 2016), *Clostridium cluster XVIII*, encompassing *C. ramosum*, involved in diet-induced obesity (Woting et al., 2014) and *C. spiroforme*, a toxin-associated disease producer, involved in rabbit colitis (Carman and Borriello, 1984).

Differentially abundant taxa, previously found in relation to rheumatoid arthritis, oral infection, intestinal inflammation or colitis, or to intestinal barrier permeability, discriminate for positivity of HLA-B27 allele, a genetic marker strongly associated with spondyloarthropathies. Among these, in HLA-B27 positive ERA patients we found *Bilophila*, a sulphite-reducing bacterium known to be involved in murine colitis (Devkota et al., 2012) and in intestinal inflammatory disorders in humans (Loubinoux et al., 2002; Rowan et al., 2009), via H_2_S production promoting intestinal inflammation. *Parvimonas* was commonly observed in periodontitis and appendicitis (Zhong et al., 2014), and *Oscillibacter* was shown to be involved in gut barrier integrity in mice (Lam et al., 2012). In HLA-B27 negative-ERA patients, we found *Haemophilus* and *Eggerthella*, recently associated with rheumatoid arthritis (Zhang et al., 2015; Chen et al., 2016). Moreover, when considering only the ERA group, in HLA-B27 positive patients we also showed enrichment of *Lactobacillus*, observed as potential arthritogenic agent via its cell wall pepetidoglycan (Severijnen et al., 1989), *Clostridium cluster XI*, that includes *C. difficile*, a well-known proinflammatory and colitis inducing-bacterium, and *Dialister*, frequently found in periodontitis and other infections (Morio et al., 2007).

Recent studies on animal models showed association between HLA-B27 allele and other different bacterial species, including *B. vulgatus* and *Prevotella spp.* (Lin et al., 2014) and *Akkermansia muciniphila* (Asquith et al., 2016). In particular, *Akkermansia* spp. was suggested as potential pro-arthritogenic bacterial genus. However, the study of Stoll and collaborators (Stoll et al., 2014) showed abundance of *Akkermansia* in a low percentage of ERA patients, but no significant association with HLA-B27 status. In our study, we did not find *Akkermansia* spp. as part of the core gut microbiota of our patients, yet we found other species correlated with HLA-B27 allele. Little is known on the geographic distribution of *Akkermansia* spp. that could be less represented in our cohorts. Overall this could suggest that other bacterial species, in absence of *Akkermansia* spp. can discriminate HLA-B27 status, and that disease biomarkers should be based on patterns of taxonomic units or biological functions, rather than on single species.

Regarding the functional contribution of microbiota profiles, by PICRUSt prediction analysis we observed distinctive functional acquisitions among JIA subgroups and HS. A core of metabolic capabilities, regarding carbohydrate, lipid, aminoacid, cofactors, and vitamins metabolism, were enriched in JIA-nERA and HS metagenomes. It is worth noting that microbiota of JIA-ERA patients is significantly enriched in functions related to cell motility, including flagellar assembly, bacterial chemotaxis and motility proteins, representing possible traits of virulence that could be associated to gut inflammation. These indications of enrichment in potentially pathogenic invasiveness-related traits in JIA-ERA metagenome could suggest a potential improved ability of microbial components to pass through the gastrointestinal barrier and migrate in other districts, also responding to nutrient gradients. Moreover, given that in mice models immunogenicity of flagellin CBir1 was observed, with consequent induction of colitis, and antibodies anti-CBir1 were found in CD patients with complicated disease (Targan et al., 2005), we cannot exclude the potential effect of flagellar-assembly proteins of some components of microbiota on host immune system of JIA.

Despite the relatively small cohort of patients in our study, the microbial profile differences in active disease and remission are corroborated by the observed intra- and inter-group distances of microbiota samples in active, remission and healthy status. As expected, our results suggest that during active disease the microbiota is strongly perturbed (major intra-group distance compared with remission). Healthy status allows a more stable microbiota ecosystem compared to disease status, as previously observed (Coyte et al., 2015). Remission is characterized by an intermediate microbial pattern, different from both active disease and healthy controls, likely resulting from a different trajectory to stable state and in which autoimmune reactivity and the microbial ecosystem are mutually shaped.

Although microbial profiles may differ in an individual-specific manner, the observed fecal microbiota dissimilarities in the same subject at different collection times, and among JIA categories and healthy controls suggest that continued perturbation and instability of the microbial ecosystem may contribute to inflammation.

Another aspect that should be more thoroughly investigated is the association between microbial profiles and pharmacologic therapies in autoimmune diseases. Recent studies, as previously observed in IBD patients, highlighted the effect of different therapies on microbiota, such as the rapid effect of enteral nutrition in the shaping of microbiota (Lionetti et al., 2005) and the dysbiosis associated with antibiotic treatment (Lewis et al., 2015), as well as the effect of protonic pomp inhibitors on reduction of bacterial richness and selection of “unhealthy” microbiota (Imhann et al., 2016). Moreover, the recent considerations suggest that the use of sulfasalazine could protect the intestinal epithelium from injury, reducing the bacterial product circulation (Rosenbaum and Asquith, 2016). Despite the reduced number of patients on single or combined therapies in the cohort, our results showed indications of different microbial profiles associated to pharmacological therapies, such as NSAIDs, immunosuppressants, and biologics, providing interesting clues on effect of such treatments on gut microbiota selection. For example, the enrichment of *Collinsella*, involved in exacerbation of joint disease (Chen et al., 2016), observed in JIA-ERA patients treated with NSAIDs in combination with sulfasalazine, results in contrast with hypothesis of Rosenbaum and Asquith (Rosenbaum and Asquith, 2016). Our results prompt future studies on larger cohorts, including untreated newly onset patients, addressing the effect of different pharmacological therapies on patients in active disease and remission, investigating how inflammation can indirectly modify microbiota, selecting differential microbial components, via mechanisms involved in epithelial barrier function and immune response and how pharmacological treatment contribute to perturb the gut microbial equilibrium compared to healthy status.

Finally, given the extreme inter-individual variability of microbiota in inflammatory and autoimmune diseases, further investigations on microbiota dynamics in different phases of disease should address the causes of perturbation and the restoration of microbial equilibrium, in order to adopt therapeutic strategies able to maintain microbial diversity and a stable state, essential for immune homeostasis and the host’s health. Research in this direction should include therapeutic strategies able to modulate the microbiota, not only with diet and probiotics, but also evaluating new therapeutic approaches, such as fecal transplantation, recently adopted in other diseases and that have shown some effectiveness, especially during active disease. Furthermore, the understanding of the microbial functional acquisition and the relationships with epithelial barrier function and host immune response could help to identify the pro-arthtritogenic contribution of the microbiota.

## Additional Information

Accession codes: Raw 454 files (.sff files) are available at the European Nucleotide Archive (http://www.ebi.ac.uk/ena/data/view/PRJEB11846) under the accession study ERP013262.

## Author Contributions

All authors were involved in drafting the article or revising it critically for important intellectual content, and all authors approved the final version to be published. Study conception and design: RC, CDF, PL, DC, MDP. Data analysis: DA, CD, MDP, MP, MS. Interpretation of data: MDP, CDF, RC, DC, PL, IP, TG, GS, AP.

## Conflict of Interest Statement

The authors declare that the research was conducted in the absence of any commercial or financial relationships that could be construed as a potential conflict of interest.

## Footnotes

1 http://www.ebi.ac.uk/ena/data/view/PRJEB11846

2 http://compmetagen.github.io/micca/
