# Supplementary material for: Alteration of Fecal Microbiota Profiles in Juvenile Idiopathic Arthritis. Associations with HLA-B27 Allele and Disease Status
Source: Front Microbiol. 2016 Oct 26;7:1703. doi: 10.3389/fmicb.2016.01703 (PMC5080347; doi:10.3389/fmicb.2016.01703)
Supplement: Supplementary file 1 [file Data_Sheet_1.pdf]

Supplementary Material

Alteration of fecal microbiota profiles in juvenile idiopathic arthritis. Associations with HLA-B27 allele and disease status.

Monica Di Paola[1], Duccio Cavalieri[2], Davide Albanese[3], Maddalena Sordo[3], Massimo Pindo[3], Claudio Donati[3], Ilaria Pagnini[4], Teresa Giani[4], Gabriele Simonini[1,4], Alessia Paladini[4], Paolo Lionetti[1], Carlotta De Filippo[5], Rolando Cimaz[1,4]

Affiliations:

[1] Department of Neuroscience, Psychology, Drug Research and Child Health, Meyer Children's Hospital, University of Florence, Viale G. Pieraccini 24, 50139 Florence, Italy.

[2] Department of Biology, University of Florence, Via Madonna del Piano 6, 50019 Sesto Fiorentino, Florence, Italy.

[3] Fondazione E. Mach, Research and Innovation Center, Via E. Mach 1, 38010 San Michele all' Adige (Trento), Italy.

[4] Rheumatology Unit, Anna Meyer Children's Hospital, University of Florence, Viale G. Pieraccini 24, 50139 Florence, Italy.

[5] Institute of Biometeorology (IBIMET), National Research Council (CNR), Via G. Caproni 8, 50145 Florence, Italy.

Correspondence to:

Rolando Cimaz, MD-PhD for Clinical part

e-mail: r.cimaz@meyer.it

Fax: +39 055 2758181

Rheumatology Unit, Anna Meyer Children's Hospital,

University of Florence, Department of Neuroscience, Psychology, Drug Research and Child Health,

Viale G. Pieraccini 24, 50139 Florence, Italy.

and

Carlotta De Filippo, PhD for Microbiological and Metagenomic part

e-mail: c.de.filippo@ibimet.cnr.it

National Research Council (CNR),

Via G. Caproni 8, 50145 Florence, Italy.

Phone +39 055 2758322

Supplementary data

Microbiota profiles and therapies

We evaluated the effect of different medical treatments on gut microbiota of JIA patients. A range of therapies are employed in the treatment of JIA, including the use of nonsteroidal anti-inflammatory drugs (NSAIDs), corticosteroids, immunosuppressive drugs, and biologics such as anti-TNFα. In severe disease, combined therapy is prescribed. Thus, we evaluated correlations between gut microbiota profiles of JIA patients and single or combined therapies. As reported in Table 1 and Supplementary Tables 1-2, in our cohorts, JIA-ERA patients were treated with NSAIDs, alone or combined with sulfasalazine/methotrexate/biologics, in different combinations. JIA-nERA patients were mainly treated with biologic drugs, such as Abatacept, a molecule blocking activation and proliferation of T cells and Etanercept, an anti-TNF alpha drug. By LEfSe analysis we observed a significantly different abundance of bacterial genera was correlated with combined therapies (Supplementary Figure 3 A-C). In particular, in JIA-ERA patients, enrichment of *Clostridium sensu stricto* was significantly correlated with NSAIDs therapy, *Dorea* and *Collinsella* was correlated with combined NSAIDs and sulfasalazine therapy, *Clostridium cluster XIVb* with combined NSAIDs and methotrexate therapy, *Slackia* and *Coprococcus* with biologic drugs, and *Alistipes* and *Oscillibacter* with combined biologics, NSAIDs and sulfasalazine therapy (Supplementary Figure 3A). In JIA-nERA patients, *Coprococcus* was significantly enriched in fecal samples of patients treated with Abatacept, while *Bacteroides* in those on Etanercept therapy (Supplementary Figure 3B). Our results showed associations among therapies and different bacterial profiles, as previously observed in IBD (Lewis, Chen et al. 2015). It is possible to hypothesize that pharmacologic therapies, acting on inflammation and the immune response, can indirectly modify microbiota, selecting differential microbial components, via mechanisms involved in epithelial barrier function and immune response. However, given the low number of patients stratified by pharmacological treatment, further studies will be needed to understand the causality between therapy, gut microbiota profiles and clinical status.

Supplementary Materials

DNA extraction, PCR amplification and pyrosequencing

For each sample, the RNAlater® (Ambion, Life Technologies) was removed from 500 mg aliquots of thawed fecal samples. Bacterial genomic DNA extraction and quality check were carried out following our previous protocol (De Filippo, Cavalieri et al. 2010), and quality-assessed by gel electrophoresis and the NanoDrop spectrophotometer (Thermo Fisher, Waltham, MA). Fecal samples were stored at -20 °C until pyrosequencing.

For each sample, we amplified the 16S rRNA gene using the special fusion primer set specific for V5-V6 hypervariable regions. The forward primer included the Lib-L primer A sequence (Roche, Branford, CT), the key sequence TCAG, the sample-specific barcode Multiplex Identifier (MID) sequence and the Forward sequence. The reverse primer comprised the Lib-L primer B sequence, the key sequence TCAG and the Reverse sequence. For each sample, a PCR mix of 25 µl was prepared containing 1X PCR buffer, 1.25 U of FastStart High Fidelity polymerase blend (Roche Life Science, Milano, Italy) and dNTPs from the FastStart High Fidelity PCR system (Roche Life Science, Milano, Italy), 0,4 µM of each primer (PRIMM, Milano, Italy) and 10 ng of gDNA. Thermal cycling consisted of initial denaturation at 95 °C for 5 minutes followed by 35 cycles of denaturation at 95 °C for 30 seconds, annealing at 58 °C for 30 seconds, and extension at 72 °C for 1 minute, with a final extension of 8 minutes at 72 °C.

Library construction and pyrosequencing

The PCR products (three replicates) of the 75 samples were analyzed by gel electrophoresis and cleaned using the AMPure XP beads kit (Beckman Coulter, Brea, CA, USA) following the manufacturer instructions; quantified via PCR using the Library quantification kit Roche 454 titanium (KAPA Biosystems, Boston, MA) and pooled in equimolar proportion in a final amplicon library.

The 454 pyrosequencing was carried out on the GS FLX+ system using the XL+ chemistry following the manufacturer recommendations.

References

De Filippo, C., D. Cavalieri, M. Di Paola, M. Ramazzotti, J. B. Poullet, S. Massart, S. Collini, G. Pieraccini and P. Lionetti (2010). Impact of diet in shaping gut microbiota revealed by a comparative study in children from Europe and rural Africa. *Proc Natl Acad Sci U S A* **107**(33): 14691-14696.

Lewis, J. D., E. Z. Chen, R. N. Baldassano, A. R. Otley, A. M. Griffiths, D. Lee, K. Bittinger, A. Bailey, E. S. Friedman, C. Hoffmann, L. Albenberg, R. Sinha, C. Compher, E. Gilroy, L. Nessel, A. Grant, C. Chehoud, H. Li, G. D. Wu and F. D. Bushman (2015). Inflammation, Antibiotics, and Diet as Environmental Stressors of the Gut Microbiome in Pediatric Crohn's Disease. *Cell Host Microbe* **18**(4): 489-500.

Supplementary Figures

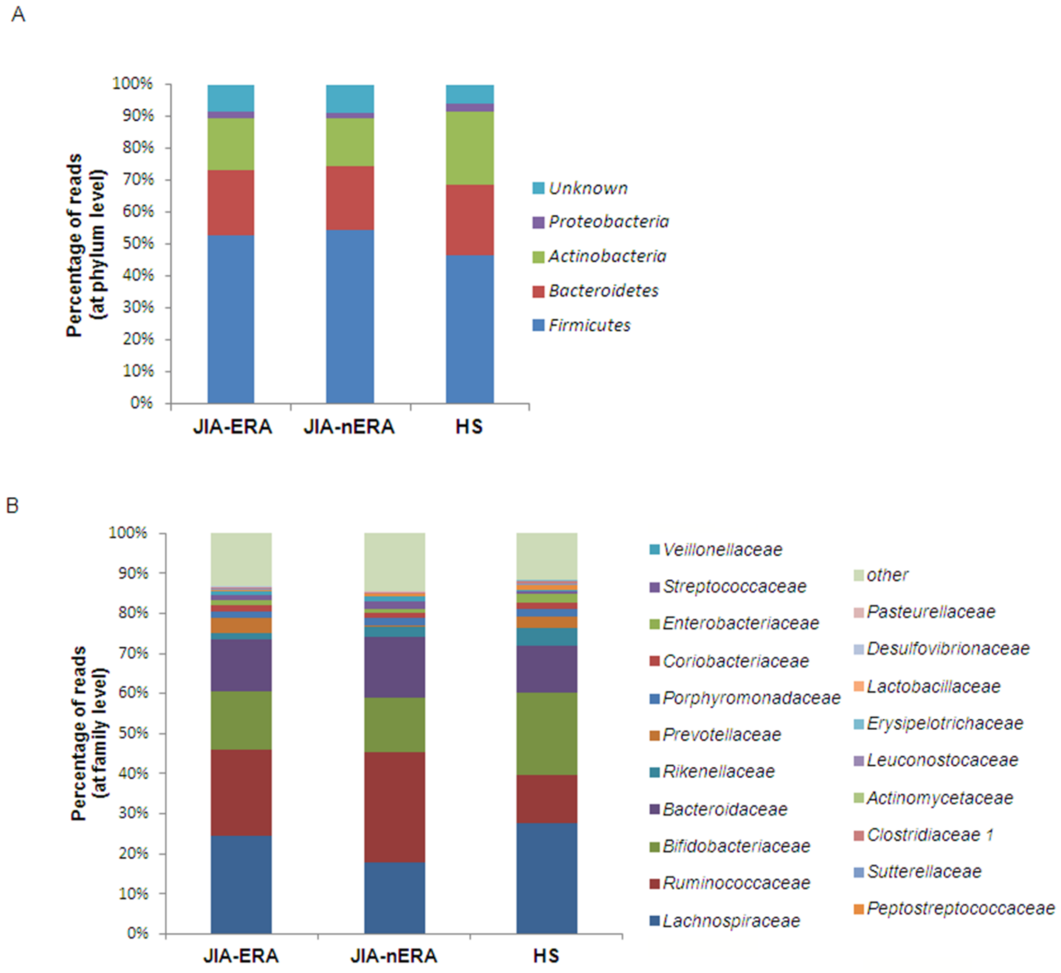

Supplementary Figure 1 Overview of fecal microbiota profiles in JIA and healthy subjects. Percentage of reads (A) of the main four phyla in JIA patients (ERA and n-ERA) and healthy subjects (HS) and (B) percentage of reads of the top 20 families in JIA patients and HS.

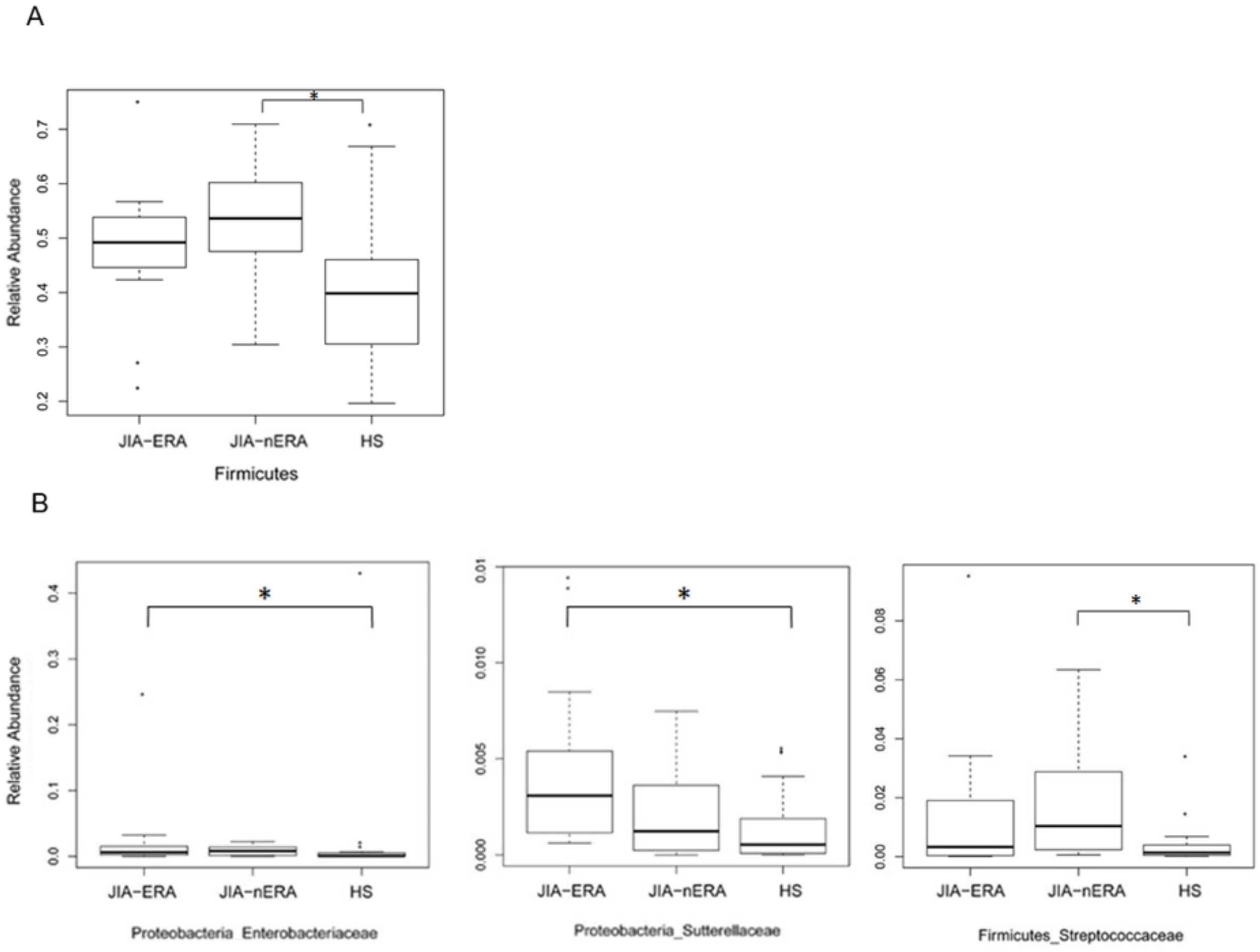

Supplementary Figure 2 Box plot of relative abundances of the statistically significant different bacterial (A) phylum and (B) families in JIA female patients compared with female HS (p-value by Wilcoxon rank-sum test, $p<0.05$).

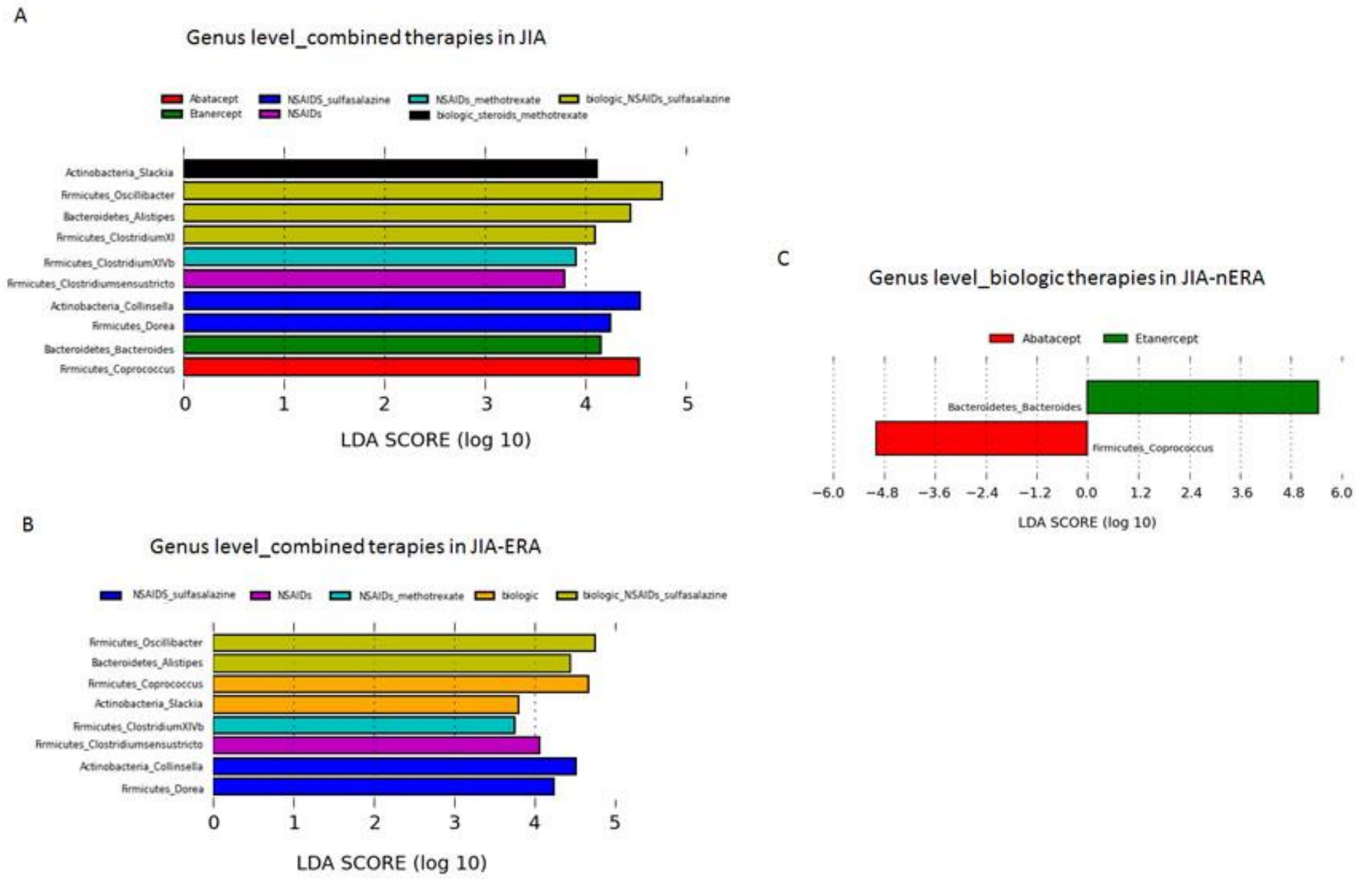

Supplementary Figure 3 Microbiota profile enrichment related to different therapies in JIA patients. LEfSe analysis performed in (A) all JIA patients, in (B) JIA-ERA, and in (C) JIA-nERA patients shows a statistically significant enrichment of bacterial genera correlated with different therapies. LEfSe results indicate a sequentially significant ranking among groups (Alpha value=0.05 for the factorial Kruskal-Wallis test among classes). The threshold for the logarithmic LDA score was 2.0.

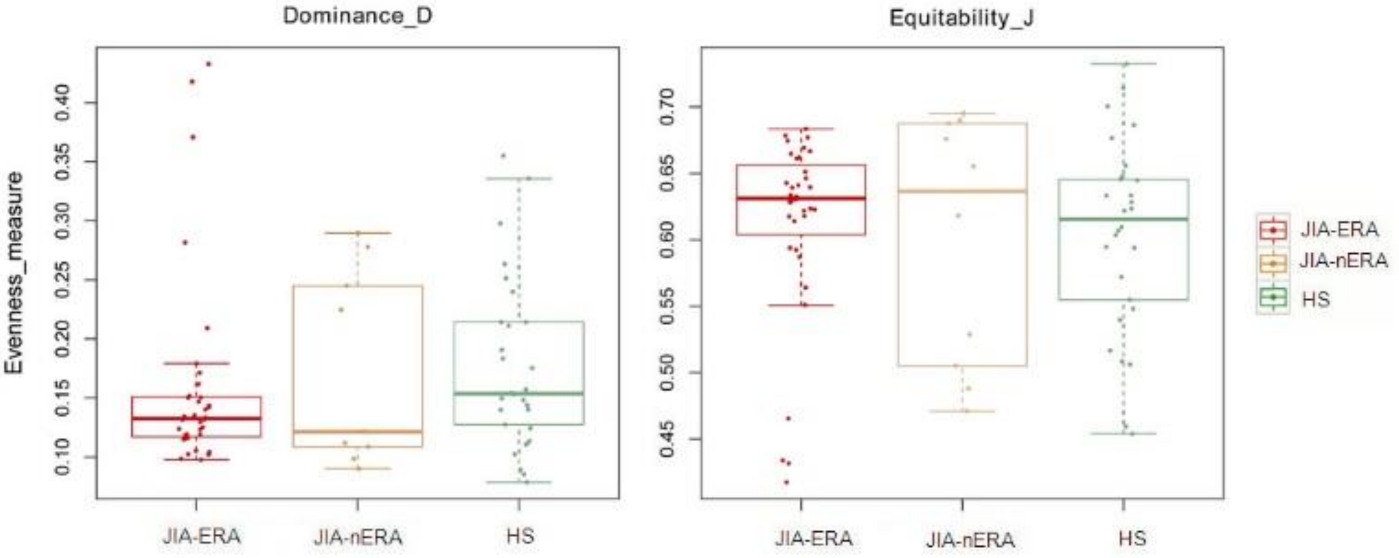

Supplementary Figure 4 Evenness indexes. Box plots of Dominance (1-Simpson index) and Equitability (Shannon index divided for logarithm of taxa number) in the three studied populations, obtained by PAST v3.12. Pairwise comparisons using the Wilcoxon rank sum test were not significant.

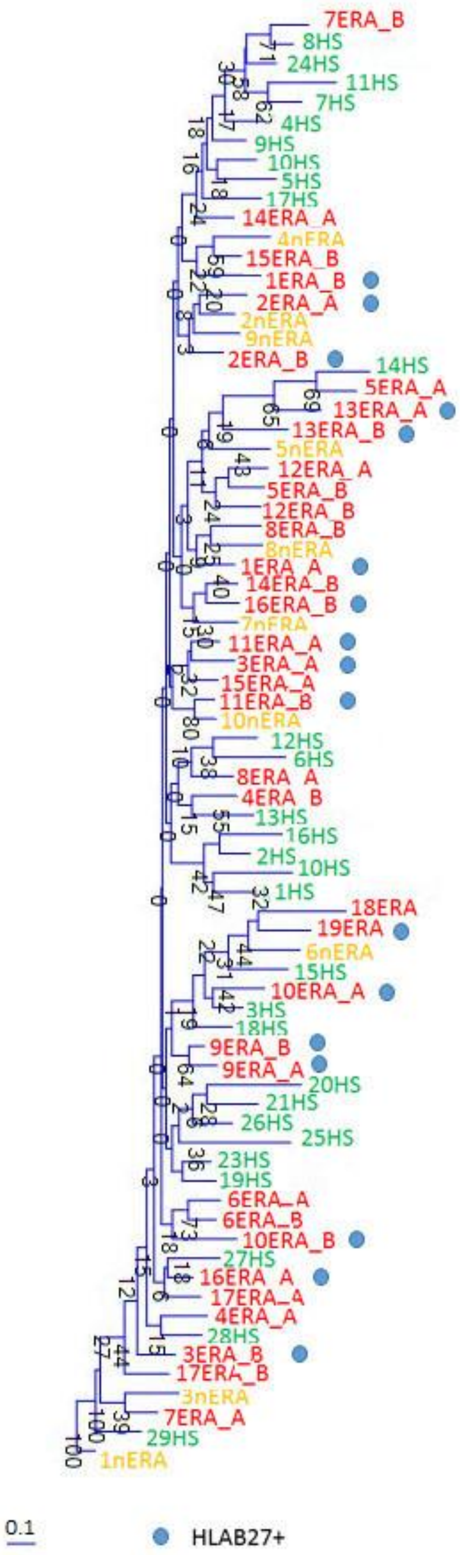

Supplementary Figure 5. Neighbor joining clustering based on Bray-Curtis distances. Numbers on branches indicate bootstrap values (9999 replicates). Scale bar indicates a distance of 0.1. Blue circles indicate HLA-B27 positive JIA-ERA patients.

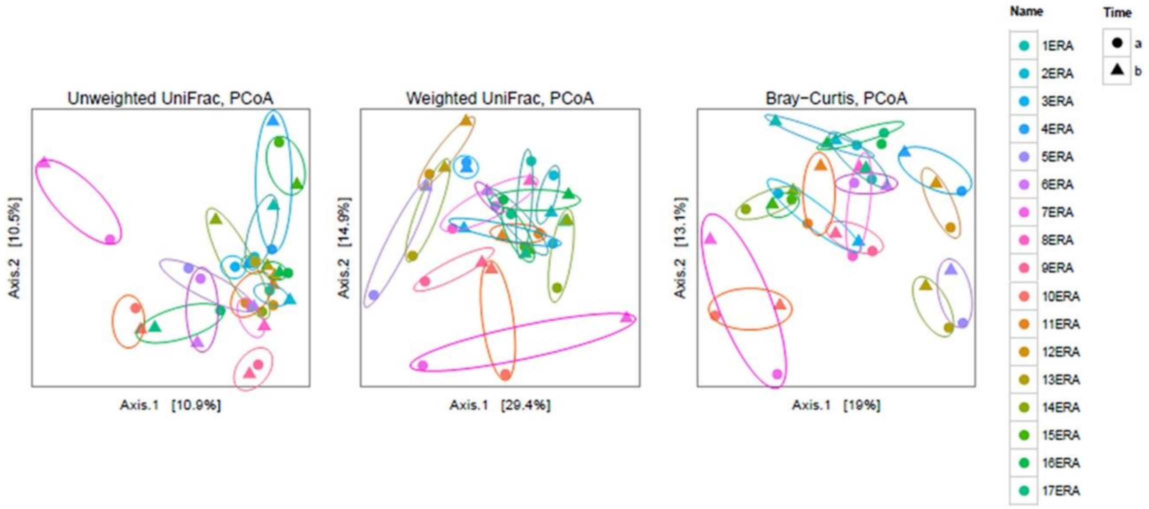

Supplementary Figure 6 Principal coordinate analysis (PCoA) derived from pairwise un-weighted and weighted UniFrac, and Bray-Curtis distances among JIA-ERA samples (A and B samples of the same JIA-ERA patient are represented as dot and triangle with same color, respectively). For each axis, in square brackets, the percent of variation explained was reported.

Supplementary Tables

Supplementary Table 1 Clinical data of JIA-ERA patients

ID patient	Sex	Age at sampling (Y= year; Mo= months)	Disease duration (Mo)	HLA B27 status	Autoantibody status	Previous or present arthritis (localization)	Previous or present Enthesitis (localization)	Active disease	Complications and comorbidities	Pain sacroiliac joint and/or spine	History of acute anterior uveitis	Pharmacologic treatment at sampling	Previous pharmacologic treatment	ESR and CRP values (ESR normal value <20 mm/hr: CRP normal value < 0.5mg/L)	Familiy History for autoimmune diseases (SpA; IBD; Uveitis; other)	Calprotectin (Normal value <50µg/g feces; abnormal value >100µg/g feces)
JIA-ERA01	M	14 Y and 4 Mo	153	positive	ANA positive; pANCA negative	Left and right hip; left and right ankle	No	No	β-thalassemia heterozygous	Y	No	Naproxen; Sulfasalazine	Naproxen	ESR normal ; CRP normal	No	Normal
JIA-ERA02	M	17 Y	114	positive	ANA negative; pANCA negative	Left hip; right knee	Left plantar and dorsal fascia	No	Pectum escavatum; diarrhoea	Y (left)	No	Naproxen	Naproxen; Sulfasalazine	ESR normal ; CRP normal	Father HLA B 27+	Normal
JIA-ERA03	M	11 Y and 8 Mo	115	positive	ANA negative; pANCA negative	Right hip; ankle	No	No	No	No	No	Ibuprofen; Naproxen; Sulfasalazine; Adalimumab	Ibuprofen; Naproxen; Sulfasalazine	ESR normal ; CRP normal	Father affected by uveitis	Normal
JIA-ERA04	M	17 Y	111	negative	ANA negative; pANCA negative	No arthritis	Heels; plantar and anterior tibial fascia; wrist flexor	No	Asthma and allergy to house dust mite	Y (bilateral); low back pain	No	Meloxicam	Sulfasalazine	ESR normal ; CRP normal	Mother affected by connective disease	Normal
JIA-ERA05	M	12 Y and 6 Mo	137	negative	ANA negative; pANCA negative	Left and right hip	Peroneal right; right plantar fascia	Y	No	Y	No	Meloxicam; Naproxen; Sulfasalazine	Naproxen; Sulfasalazine	ESR normal ; CRP normal	No	Normal

JIA-ERA06	F	16 Y and 3 Mo	108	negative	ANA negative; pANCA negative	Left and right knee; right temporomandibular joint; proximal interphalangeal 1st-2nd-3rd-4th; metacarpal phalangeal 1st-2nd-3rd right hand; metatarsal phalangeal 3rd right foot; metatarsal phalangeal 1st-3rd left foot; proximal interphalangeal 2nd toe of right foot; proximal interphalangeal 1st-2nd-3rd-4th finger of left hand	Bilateral plantar fascia;	Y	No	Y	No	Naproxen; Sulfasalazine	Naproxen; Sulfasalazine	ESR normal ; CRP normal	Mother affected by coeliac disease and rheumatoid arthritis	Normal
JIA-ERA07	M	13 Y	78	negative	ANA positive; pANCA positive	Left knee; right subtalar	No	Y	Pectum escavatum, cryptorchidism	Y (right)	No	Prednisone; Naproxen; Sulfasalazine	Prednisone; Naproxene; Flurbiprofen; Sulfasalazine;	ESR abnormal; CRP normal	No	Normal
JIA-ERA08	F	14 Y	94	negative	ANA positive; pANCA negative	Hips; elbows; right shoulder; right knee; right ankle; right temporomandibular joint	No	No	Celiac disease; Osteoporosis	No	No	Naproxen; Methotrexate; Meloxicam; Etanercept	Naproxene; Methotrexate; Meloxicam; Etanercept	ESR abnormal; CRP normal	Mother affected by coeliac disease	Normal
JIA-ERA09	F	15 Y and 2 Mo	96	positive	ANA positive; pANCA negative	Right and left ankles; right temporomandibular joint; right wrist ; metacarpal phalangeal of 1st-2nd-3rd-4th finger of left and right hand; metatarsal phalangeal of 1st-2nd-3rd toe of left foot; right knee	Right wrist flexors; Bilateral plantar fascia	No	Breast fibroadenoma; obesity; cortical cataract, allergic asthma	Y (bilateral)	Y	Prednisone; Methotrexate; Indomethacin; Abatacept; Indomethacin	Prednisone; Methotrexate; Indoxen, Infliximab; Meloxicam; Adalimumab	ESR normal ; CRP normal	Maternal aunt affected by Crohn's disease	Normal
JIA-ERA10	M	15 Y and 6 Mo	178	positive	ANA negative; pANCA negative	Right hip; left knee		No	No		No	Indomethacin; Naproxen	Naproxen	ESR normal ; CRP normal	No	Abnormal

JIA-ERA11	F	9 Y and 9 Mo	106	positive	ANA positive; pANCA neg	Ankles; cervical spine; temporomandibular joint; right hip; right knee; right shoulder; right tarsus; left metatarsal phalangeal	Left achilles	Y	No	Y (bilateral)	No	Naproxen; Methotrexate; Adalimumab	Naproxen; Methotrexate	ESR normal ; CRP normal	No	
JIA-ERA12	M	17 and 8 Mo	126	negative	ANA negative; pANCA negative	Metacarpal phalangeal of 1st-2nd-5th finger of right hand; metacarpal phalangeal of 1st-2nd-3rd fingers of left hand; proximal interphalangeal of 1st-2nd finger of right and left hand; wrist; right ankle; knees	Achilles	No	No	Y (bilateral)	No	Naproxen	Sulfasalazine; Methotrexate; Naproxen	ESR normal ; CRP normal	No	Normal
JIA-ERA13	F	15 Y and 5 Mo	163	positive	AN Anegative; pANCA negative	Right ankle; metatarsal phalangeal and proximal interphalangeal of 1st-2nd-3rd finger; left foot; right hip; ankles; metatarsal phalangeal of 1st toe of right foot	Right and left achilles	No	No	No	No	Naproxen	Sulfasalazine; Naproxen	ESR normal ; CRP normal	Not available (adopted child of Indian origin)	Normal
JIA-ERA14	M	10 Y and 2 Mo	84	negative	ANA negative; pANCA negative	Right knee; wrist; metacarpal phalangeal of left foot; right ankle;	Achilles and bilateral heels	No	Hypothyroidism and obesity	Y (bilateral and Lumbosacral spine)	No	Etanercept	Ibuprofen; Naproxen	ESR abnormal; CRP normal	No	Abnormal
JIA-ERA15	M	14 Y and 3 Mo	82	negative	ANA positive; pANCA negative	Right knee; left shoulder; ankles; metatarsal phalangeal of 1st finger	Bilateral plantar fascia	No	Early puberty	Y (bilateral and Lumbosacral spine)	No	Meloxicam; Naproxene; Methotrexate	Meloxicam; Naproxen; Methotrexate	ESR normal ; CRP normal	No	Normal
JIA-ERA16	M	9 Y	96	positive	ANA negative; pANCA negative	Hips	No	No	Epigastric hernia; hypospadia	No	No	Naproxene	Naproxen	ESR normal ; CRP normal	No	Normal
JIA-ERA17	M	13 Y and 7 Mo	86	negative	ANA negative; pANCA negative	Wrist	No	No	No	Y (bilateral)	No	Prednisone; Naproxen; Sulfasazine	Naproxen; Sulfasazine;	ESR normal ; CRP normal	mother affected by psoriasis ; cousin affected by Crohn's disease	

JIA-ERA18	F	17 Y and 8 Mo	110	negative	ANA negative; pANCA negative	no	No	No	No	No	No	Naproxen; Sulfasazine;	Naproxen; Sulfasazine;	ESR normal ; CRP normal	No	Normal
JIA-ERA19	M	12 Y	123	positive	ANA negative; pANCA negative	Hips; 5th toe of right foot; left knee; ankle	Right achilles	No	Morphea at left knee	Y (bilateral)	No	Prednisone; Naproxen; Sulfasalazine; Etanercept	Prednisone; Naproxen	ESR normal ; CRP normal	No	Normal

Supplementary Table 2 Clinical data of JIA-nERA patients

ID patient	sex	Age at sampling (Y= year; Mo= months)	Disease duration (Mo)	Complications and comorbidities	Previous pharmacologic treatment	Pharmacologic treatment at sampling	Active disease (Y=yes)	Calprotectin (Normal value <50µg/g feces; abnormal value >100µg/g feces)
JIA-n ERA01	F	17 Y	130	No	NSAIDs; Methotrexate; Prednisone	Etanercept	Y	Normal
JIA-n ERA02	F	7 Y and 1 Mo	34	No	NSAIDs; Methotrexate	Etanercept	Y	Normal
JIA-n ERA03	F	2 Y and 2 Mo	23	No	NSAIDs; Methotrexate; Prednisone	Etanercept	No	Abnormal
JIA-n ERA04	F	15 Y and 8 Mo	138	uveitis; conjunctivitis	NSAIDs; Methotrexate; anti-TNFα (Etanercept; Adalimumab); Prednisone	Abatacept	No	Normal
JIA-n ERA05	F	14 Y and 3 Mo	150	uveitis	NSAIDs; Methotrexate; anti-TNFα (Etanercept; Adalimumab); Prednisone	Abatacept	No	Normal
JIA-n ERA06	F	10 Y	98	uveitis	NSAIDs; Methotrexate; Prednisone	Etanercept	No	Normal
JIA-n ERA07	F	5 Y and 9 Mo	26	uveitis	NSAIDs; Methotrexate; anti-TNFα (Etanercept; Adalimumab); Prednisone	Abatacept	No	Normal
JIA-n ERA08	F	12 Y and 6 Mo	109	uveitis	NSAIDs; Methotrexate; anti-TNFα (Etanercept; Adalimumab); Prednisone	Abatacept	Y	Normal
JIA-n ERA09	F	11 Y and 6 Mo	96	uveitis	NSAIDs; Methotrexate; anti-TNFα (Adalimumab)	Abatacept	No	Normal
JIA-n ERA10	F	2 Y and 10 Mo	3	uveitis	NSAIDs	NSAIDs	Y	Abnormal

Supplementary Table 3. P-value of alpha diversity (Pairwise Wilcoxon rank-sum test), and beta diversity (PERMANOVA), as referred in Fig. 3.

Alpha diversity, Pairwise Wilcoxon rank-sum test, p value, FDR correction

Observed	JIA-ERA	JIA-nERA
JIA-nERA	0.1394	-
HS	0.0034	0.0036

Chao 1	JIA-ERA	JIA-nERA
JIA-nERA	0.1905	-
HS	0.0049	0.0049

Shannon	JIA-ERA	JIA-nERA
JIA-nERA	0.55	-
HS	0.32	0.32

Beta diversity, PERMANOVA

	F	R2	p-val
unweighted Unifrac ADONIS	2.8594	0.0736	0.0001
weighted Unifrac ADONIS	2.4524	0.0638	0.0034
Bray-Curtis ADONIS	2.7321	0.0705	0.0001

Supplementary Table 4. P-value of Wilcoxon rank-sum test obtained by comparison of intra-group and inter-group distances calculated by Unweighted UniFrac between samples collected during active disease and in remission (from JIA-ERA and JIA-nERA patients). In red, intra-group distances, as in Figure 4B, are indicated.

Var1	Var2	p-value
JIA- ERA Acute vs. JIA- ERA Remission	JIA- ERA Acute vs. JIA- ERA Acute	0.733314
JIA- ERA Acute vs. JIA- nERA Acute	JIA- ERA Acute vs. JIA- ERA Acute	0.419741
JIA- ERA Acute vs. JIA- nERA Remission	JIA- ERA Acute vs. JIA- ERA Acute	0.730393
JIA- ERA Acute vs. Healthy	JIA- ERA Acute vs. JIA- ERA Acute	0.8806
JIA- ERA Remission vs. JIA- ERA Remission	JIA- ERA Acute vs. JIA- ERA Acute	0.218291
JIA- ERA Remission vs. JIA- nERA Acute	JIA- ERA Acute vs. JIA- ERA Acute	0.227223
JIA- ERA Remission vs. JIA- nERA Remission	JIA- ERA Acute vs. JIA- ERA Acute	0.767438
JIA- ERA Remission vs. Healthy	JIA- ERA Acute vs. JIA- ERA Acute	0.579375
JIA- nERA Acute vs. JIA- nERA Acute	JIA- ERA Acute vs. JIA- ERA Acute	0.361524
JIA- nERA Acute vs. JIA- nERA Remission	JIA- ERA Acute vs. JIA- ERA Acute	0.790175
JIA- nERA Acute vs. Healthy	JIA- ERA Acute vs. JIA- ERA Acute	0.703927
JIA- nERA Remission vs. JIA- nERA Remission	JIA- ERA Acute vs. JIA- ERA Acute	0.790175
JIA- nERA Remission vs. Healthy	JIA- ERA Acute vs. JIA- ERA Acute	0.043657
Healthy vs. Healthy	JIA- ERA Acute vs. JIA- ERA Acute	0.018408
JIA- ERA Acute vs. JIA- nERA Acute	JIA- ERA Acute vs. JIA- ERA Remission	0.443223
JIA- ERA Acute vs. JIA- nERA Remission	JIA- ERA Acute vs. JIA- ERA Remission	0.120679
JIA- ERA Acute vs. Healthy	JIA- ERA Acute vs. JIA- ERA Remission	0.20505
JIA- ERA Remission vs. JIA- ERA Remission	JIA- ERA Acute vs. JIA- ERA Remission	0.037965
JIA- ERA Remission vs. JIA- nERA Acute	JIA- ERA Acute vs. JIA- ERA Remission	0.247023
JIA- ERA Remission vs. JIA- nERA Remission	JIA- ERA Acute vs. JIA- ERA Remission	0.038498
JIA- ERA Remission vs. Healthy	JIA- ERA Acute vs. JIA- ERA Remission	0.820721
JIA- nERA Acute vs. JIA- nERA Acute	JIA- ERA Acute vs. JIA- ERA Remission	0.528502
JIA- nERA Acute vs. JIA- nERA Remission	JIA- ERA Acute vs. JIA- ERA Remission	0.935549
JIA- nERA Acute vs. Healthy	JIA- ERA Acute vs. JIA- ERA Remission	0.829657

JIA- nERA Remission vs. JIA- nERA Remission	JIA- ERA Acute vs. JIA- ERA Remission	0.655632
JIA- nERA Remission vs. Healthy	JIA- ERA Acute vs. JIA- ERA Remission	1.81E-10
Healthy vs. Healthy	JIA- ERA Acute vs. JIA- ERA Remission	3.10E-07
JIA- ERA Acute vs. JIA- nERA Remission	JIA- ERA Acute vs. JIA- nERA Acute	0.028187
JIA- ERA Acute vs. Healthy	JIA- ERA Acute vs. JIA- nERA Acute	0.123193
JIA- ERA Remission vs. JIA- ERA Remission	JIA- ERA Acute vs. JIA- nERA Acute	0.937747
JIA- ERA Remission vs. JIA- nERA Acute	JIA- ERA Acute vs. JIA- nERA Acute	0.815572
JIA- ERA Remission vs. JIA- nERA Remission	JIA- ERA Acute vs. JIA- nERA Acute	0.022784
JIA- ERA Remission vs. Healthy	JIA- ERA Acute vs. JIA- nERA Acute	0.408746
JIA- nERA Acute vs. JIA- nERA Acute	JIA- ERA Acute vs. JIA- nERA Acute	0.839805
JIA- nERA Acute vs. JIA- nERA Remission	JIA- ERA Acute vs. JIA- nERA Acute	0.417035
JIA- nERA Acute vs. Healthy	JIA- ERA Acute vs. JIA- nERA Acute	0.209062
JIA- nERA Remission vs. JIA- nERA Remission	JIA- ERA Acute vs. JIA- nERA Acute	0.363253
JIA- nERA Remission vs. Healthy	JIA- ERA Acute vs. JIA- nERA Acute	5.25E-06
Healthy vs. Healthy	JIA- ERA Acute vs. JIA- nERA Acute	0.198885
JIA- ERA Acute vs. Healthy	JIA- ERA Acute vs. JIA- nERA Remission	0.560249
JIA- ERA Remission vs. JIA- ERA Remission	JIA- ERA Acute vs. JIA- nERA Remission	0.001228
JIA- ERA Remission vs. JIA- nERA Acute	JIA- ERA Acute vs. JIA- nERA Remission	0.000975
JIA- ERA Remission vs. JIA- nERA Remission	JIA- ERA Acute vs. JIA- nERA Remission	0.733314
JIA- ERA Remission vs. Healthy	JIA- ERA Acute vs. JIA- nERA Remission	0.028187
JIA- nERA Acute vs. JIA- nERA Acute	JIA- ERA Acute vs. JIA- nERA Remission	0.059789
JIA- nERA Acute vs. JIA- nERA Remission	JIA- ERA Acute vs. JIA- nERA Remission	0.198885
JIA- nERA Acute vs. Healthy	JIA- ERA Acute vs. JIA- nERA Remission	0.108477
JIA- nERA Remission vs. JIA- nERA Remission	JIA- ERA Acute vs. JIA- nERA Remission	0.743686
JIA- nERA Remission vs. Healthy	JIA- ERA Acute vs. JIA- nERA Remission	0.012183
Healthy vs. Healthy	JIA- ERA Acute vs. JIA- nERA Remission	8.31E-07
JIA- ERA Remission vs. JIA- ERA Remission	JIA- ERA Acute vs. Healthy	6.91E-05
JIA- ERA Remission vs. JIA- nERA Acute	JIA- ERA Acute vs. Healthy	0.005065
JIA- ERA Remission vs. JIA- nERA Remission	JIA- ERA Acute vs. Healthy	0.603377
JIA- ERA Remission vs. Healthy	JIA- ERA Acute vs. Healthy	0.028509
JIA- nERA Acute vs. JIA- nERA Acute	JIA- ERA Acute vs. Healthy	0.312574

JIA- nERA Acute vs. JIA- nERA Remission	JIA- ERA Acute vs. Healthy	0.552604
JIA- nERA Acute vs. Healthy	JIA- ERA Acute vs. Healthy	0.316526
JIA- nERA Remission vs. JIA- nERA Remission	JIA- ERA Acute vs. Healthy	0.968768
JIA- nERA Remission vs. Healthy	JIA- ERA Acute vs. Healthy	1.79E-06
Healthy vs. Healthy	JIA- ERA Acute vs. Healthy	2.77E-12
JIA- ERA Remission vs. JIA- nERA Acute	JIA- ERA Remission vs. JIA- ERA Remission	0.730393
JIA- ERA Remission vs. JIA- nERA Remission	JIA- ERA Remission vs. JIA- ERA Remission	4.21E-07
JIA- ERA Remission vs. Healthy	JIA- ERA Remission vs. JIA- ERA Remission	0.004257
JIA- nERA Acute vs. JIA- nERA Acute	JIA- ERA Remission vs. JIA- ERA Remission	0.790175
JIA- nERA Acute vs. JIA- nERA Remission	JIA- ERA Remission vs. JIA- ERA Remission	0.316526
JIA- nERA Acute vs. Healthy	JIA- ERA Remission vs. JIA- ERA Remission	0.026438
JIA- nERA Remission vs. JIA- nERA Remission	JIA- ERA Remission vs. JIA- ERA Remission	0.227223
JIA- nERA Remission vs. Healthy	JIA- ERA Remission vs. JIA- ERA Remission	2.33E-23
Healthy vs. Healthy	JIA- ERA Remission vs. JIA- ERA Remission	0.000387
JIA- ERA Remission vs. JIA- nERA Remission	JIA- ERA Remission vs. JIA- nERA Acute	2.29E-05
JIA- ERA Remission vs. Healthy	JIA- ERA Remission vs. JIA- nERA Acute	0.137703
JIA- nERA Acute vs. JIA- nERA Acute	JIA- ERA Remission vs. JIA- nERA Acute	0.641632
JIA- nERA Acute vs. JIA- nERA Remission	JIA- ERA Remission vs. JIA- nERA Acute	0.349009
JIA- nERA Acute vs. Healthy	JIA- ERA Remission vs. JIA- nERA Acute	0.023548
JIA- nERA Remission vs. JIA- nERA Remission	JIA- ERA Remission vs. JIA- nERA Acute	0.355965
JIA- nERA Remission vs. Healthy	JIA- ERA Remission vs. JIA- nERA Acute	1.60E-16
Healthy vs. Healthy	JIA- ERA Remission vs. JIA- nERA Acute	0.002802
JIA- ERA Remission vs. Healthy	JIA- ERA Remission vs. JIA- nERA Remission	0.001054
JIA- nERA Acute vs. JIA- nERA Acute	JIA- ERA Remission vs. JIA- nERA Remission	0.120679
JIA- nERA Acute vs. JIA- nERA Remission	JIA- ERA Remission vs. JIA- nERA Remission	0.19484
JIA- nERA Acute vs. Healthy	JIA- ERA Remission vs. JIA- nERA Remission	0.059789
JIA- nERA Remission vs. JIA- nERA Remission	JIA- ERA Remission vs. JIA- nERA Remission	0.820721
JIA- nERA Remission vs. Healthy	JIA- ERA Remission vs. JIA- nERA Remission	8.31E-07
Healthy vs. Healthy	JIA- ERA Remission vs. JIA- nERA Remission	2.34E-15
JIA- nERA Acute vs. JIA- nERA Acute	JIA- ERA Remission vs. Healthy	0.454694
JIA- nERA Acute vs. JIA- nERA Remission	JIA- ERA Remission vs. Healthy	0.920188

JIA- nERA Acute vs. Healthy	JIA- ERA Remission vs. Healthy	0.603377
JIA- nERA Remission vs. JIA- nERA Remission	JIA- ERA Remission vs. Healthy	0.605789
JIA- nERA Remission vs. Healthy	JIA- ERA Remission vs. Healthy	4.49E-20
Healthy vs. Healthy	JIA- ERA Remission vs. Healthy	6.88E-14
JIA- nERA Acute vs. JIA- nERA Remission	JIA- nERA Acute vs. JIA- nERA Acute	0.444345
JIA- nERA Acute vs. Healthy	JIA- nERA Acute vs. JIA- nERA Acute	0.272081
JIA- nERA Remission vs. JIA- nERA Remission	JIA- nERA Acute vs. JIA- nERA Acute	0.624927
JIA- nERA Remission vs. Healthy	JIA- nERA Acute vs. JIA- nERA Acute	0.01164
Healthy vs. Healthy	JIA- nERA Acute vs. JIA- nERA Acute	0.758299
JIA- nERA Acute vs. Healthy	JIA- nERA Acute vs. JIA- nERA Remission	0.730393
JIA- nERA Remission vs. JIA- nERA Remission	JIA- nERA Acute vs. JIA- nERA Remission	0.722276
JIA- nERA Remission vs. Healthy	JIA- nERA Acute vs. JIA- nERA Remission	0.000219
Healthy vs. Healthy	JIA- nERA Acute vs. JIA- nERA Remission	0.023548
JIA- nERA Remission vs. JIA- nERA Remission	JIA- nERA Acute vs. Healthy	0.721634
JIA- nERA Remission vs. Healthy	JIA- nERA Acute vs. Healthy	3.95E-10
Healthy vs. Healthy	JIA- nERA Acute vs. Healthy	2.57E-06
JIA- nERA Remission vs. Healthy	JIA- nERA Remission vs. JIA- nERA Remission	0.060385
Healthy vs. Healthy	JIA- nERA Remission vs. JIA- nERA Remission	0.028187
Healthy vs. Healthy	JIA- nERA Remission vs. Healthy	4.84E-35

Supplementary Table 5. PICRUSt analysis output table of KEGG categories for each sample, available in data sheet .excel.
